# *Eriocomavaldesii*, a new species from México (Poaceae, Stipeae)

**DOI:** 10.3897/phytokeys.139.47373

**Published:** 2020-01-27

**Authors:** Paul M. Peterson, Konstantin Romaschenko, Robert J. Soreng, Jesus Valdés Reyna

**Affiliations:** 1 Department of Botany MRC-166, National Museum of Natural History, Smithsonian Institution, Washington, DC 20013-7012, USA National Museum of Natural History, Smithsonian Institution Washington United States of America; 2 Departamento de Botánica, Universidad Autónoma Agraria Antonio Narro, Saltillo, C.P. 25315, México Universidad Autónoma Agraria Antonio Narro Saltillo Mexico

**Keywords:** *Eriocoma*, grasses, Poaceae, Stipeae, taxonomy

## Abstract

*Eriocomavaldesii***sp. nov.**, is described and illustrated. The new species was found growing on calcareous rocky slopes and hillsides between 1700–2721 m in Coahuila, Nuevo León, San Luis Potosí, and Tamaulipas. The new species is morphologically similar to *Eriocomalobata* but differs in having ligules (2–) 4.5–8.5 mm long with acute to narrowly acute and lacerate apices and florets with a sharp-pointed callus. In addition, we include a key to the species of *Eriocoma* in northeastern México.

## Abstract (es)

Se describe e ilustra una nueva especie, *Eriocomavaldesii***sp. nov.** La nueva especie se encontró creciendo en laderas calcáreas rocosas y laderas entre 1700–2721 m en Coahuila, Nuevo León, San Luis Potosí y Tamaulipas. La nueva especie es morfológicamente similar a *Eriocomalobata*, pero difiere en tener lígulas de (2–) 4.5–8.5 mm de largo con ápices y flósculos agudos a estrechamente agudos y lacerados con un callo puntiagudo. Además, incluimos una clave para las especies de *Eriocoma* en el noreste de México.

## Introduction

The tribe Stipeae Dumort. comprises temperate, cool-season (C_3_) grasses that generally occupy somewhat moist to predominantly dry temperate steppe communities worldwide. They represent an ecologically and morphologically specialized lineage within the subfamily Pooideae including approximately 527 species in 33 genera and a single hybrid genus (Tzvelev 1977; Watson and Dallwitz 1992; Barkworth 2007; Romaschenko et al. 2008, 2010, 2011, 2012, 2013, 2014; Soreng et al. 2015, 2017; Peterson et al. 2019). Grasses in this tribe have spikelets with a single floret without a rachilla extension, disarticulation above the glumes, round to pointed well-developed calluses, membranous to coriaceous or indurate lemmas usually with a conspicuous terminal awn, two or three linear-elliptic faintly-vascularized lodicules, and indurate caryopses with a linear hilum (Tzvelev 1977; Clayton and Renvoize 1986; Watson and Dallwitz 1992, Barkworth 2007; Everett et al. 2009; Romaschenko et al. 2012; Clayton et al. 2016; Peterson et al. 2019).

*Eriocoma* Nutt., recently resurrected to replace *Achnatherum* P. Beauv. for most of the American species, consists of 27 species in North America (Canada, México, and USA), and is characterized by having a maize-like lemma epidermal pattern along with the same features mentioned above for the tribe (Romaschenko et al. 2012, 2014; Peterson et al. 2019). Within México, 7–11 species of *Eriocoma* have been reported (Espejo Serna et al. 2000; Dávila et al. 2018; Sánchez-Ken 2018). The remaining six species of *Achnatherum* in México were placed in *Pseudoeriocoma* Romasch., P.M. Peterson & Soreng, and these all have woody, sometimes scandent bamboo-like culms with ramified branching at the middle and upper nodes (Peterson et al. 2019).

Thirty years ago, in September 1989, Penelope Sue Hoge along with Mary E. Barkworth and Jesus Valdés Reyna gathered material from a new species (referred to as “*valdesii*”) near Estación Carneros, Coahuila. Hoge (1992) was working on a master’s thesis with Barkworth at Utah State University coined and placed “*valdesii*” as a subspecies of *Stipaalta* Swallen. We (PMP, KR & JVR) visited the same locality in 2012 and 2019, and gathered more material from “*valdesii*” to include in our molecular DNA sequence studies investigating the phylogeny of *Eriocoma* and *Pseudoeriocoma* (Valdés Reyna et al. 2013). Based on our unpublished phylogeny of DNA nuclear/plastid sequences and morphological study we describe “*valdesii*” as a new species of *Eriocoma*. In addition, we include a key to the species of *Eriocoma* that we have seen vouchers of from northeastern México (Coahuila, Nuevo León, San Luis Potosí, Tamaulipas).

## Taxonomy

### Eriocoma valdesii

Taxon classificationPlantaePoalesStipeae

Hoge ex Romasch., P.M. Peterson & Soreng sp. nov.

BEA8CD78-6EDF-5550-92B6-F41A09801226

urn:lsid:ipni.org:names:77204848-1

Fig. 1A–M

#### Type.

México, Coahuila, Municipio de Saltillo, 2 km above Estación Carneros just below microondas [25.12306N, 101.11828W], 2270 m, 13 Sep 2012, *Peterson, Romaschenko & Valdés Reyna 24469* (holotype: US-3741901!; isotypes: ANSM!, US-3741902!).

#### Diagnosis.

Differing from *Eriocomalobata* (Swallen) Romasch. in having ligules (2–) 4.5–8.5 mm long with acute to narrowly acute and lacerate apices (verses ligules ≤ 1.5 mm long with truncate apices) and florets with a sharp-pointed callus (verses florets with a blunt callus) [Swallen 1933; Barkworth 2007; Valdés Reyna 2015].

#### Description.

***Perennials***, cespitose, without rhizomes. ***Culms*** 60–130 cm tall, erect, unbranched above, 2–4 mm in diameter near the base, nodes 3–4 below the inflorescence, glabrous. ***Leaf sheaths*** shorter than the internodes, glabrous, older ones dorsally flattened below; **collar** glabrous or sparsely pubescent; ***ligules*** (2–) 4.5–8.5 mm long, strongly asymmetrical, membranous, margins decurrent, often with small hairs above, the hairs less than 1 mm long, apex acute to narrowly acute, lacerate; ***blades*** (15–) 25–50 (–60) cm long, (2–) 3–5 mm wide, flat to involute, glabrous, smooth below, scabrous above and along margins. ***Panicles*** 12–35 cm long, 1–2 cm wide, narrow and contracted; ***branches*** 1–6.5 cm long, ascending, straight and tightly appressed. ***Spikelets*** 8–13 mm long, usually lanceolate, subterete, rarely laterally compressed, with one fertile floret without rachilla extension, disarticulation above the glumes; ***glumes*** 8–13 mm long, longer than the florets, membranous, mostly hyaline above, 3-veined, unawned, apex long acuminate; ***lower glumes*** 10–13 mm long; ***upper glumes*** 8–12 mm long; ***florets*** (including the callus) 6–7.5 mm long, terete, fusiform, straminious to light brownish; **callus** about 1–1.4 mm long, densely bearded with hairs up to 1.5 mm long, the base sharp-pointed and slightly curved; ***lemmas*** coriaceous, indurate, evenly hairy, the hairs 1–1.4 (–2) mm long, apex 2-lobed, the lobes about 0.2–0.5 mm long; ***lemmatal awns*** 12–24 mm long, 1 or 2-geniculate, the lowest one or two segments twisted and short hairy (sometimes only visible in young material), the hairs less than 1 mm long, upper segment scabrous; ***paleas*** 3.2–5 mm long, shorter than the lemma, hairy, 2-veined, veins not prolonged; ***stamens*** 3, ***anthers*** (2.6–) 3–3.3 mm long, dehiscent, penicillate; ***lodicules*** 2 or 3, about 1.2–1.5 mm long, narrow-elliptic; stigmas 2. ***Caryopses*** 4–6 mm long, fusiform, pericarp adherent, hilum linear.

**Figure 1.**
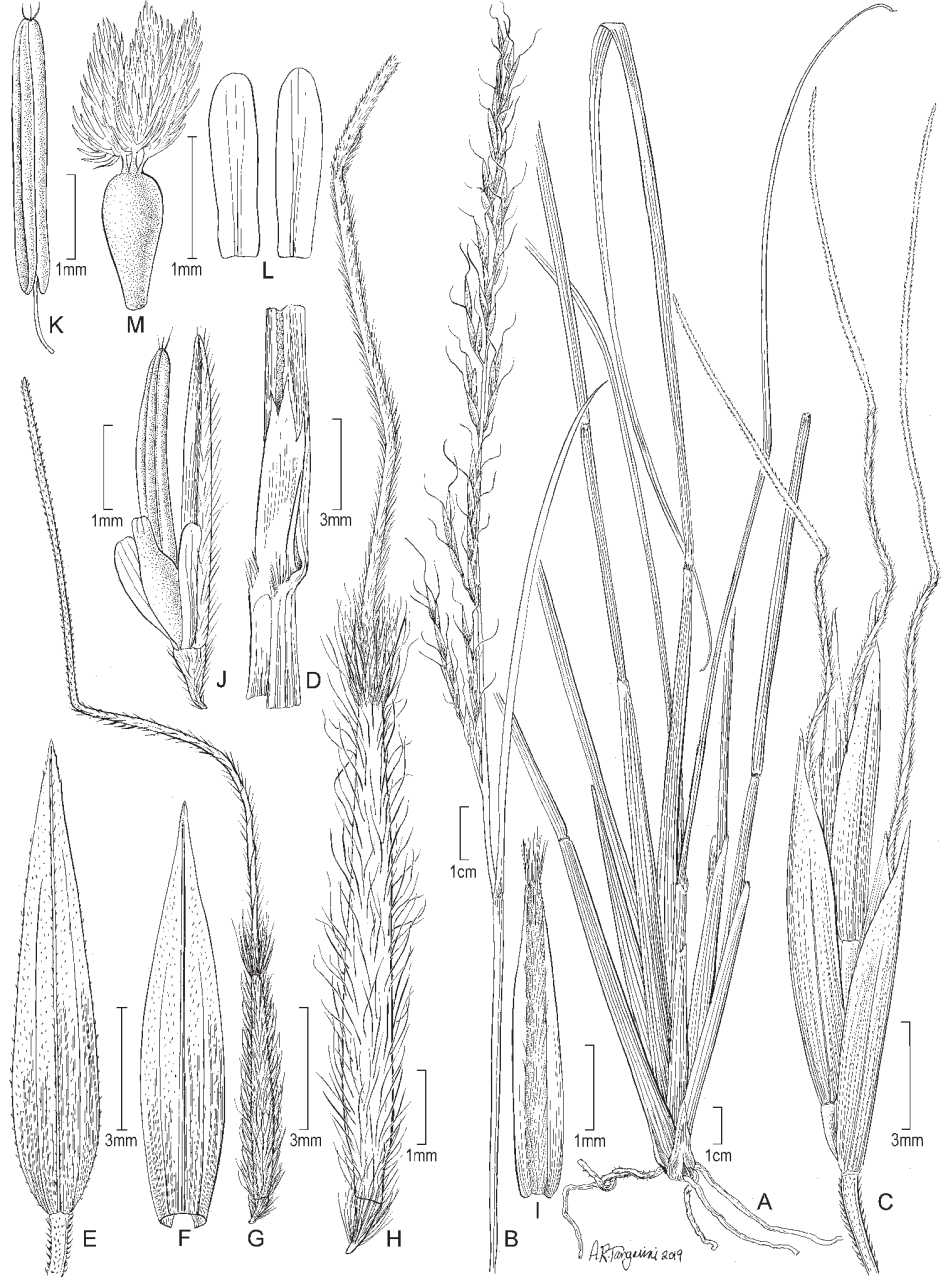
*Eriocomavaldesii*. **A** Habit **B** culm and panicle **C** panicle branch **D** sheath, ligule, and blade **E** lower glume **F** upper glume **G** floret **H** floret, enlarged **I** palea **J** palea, lodicules, ovary, and stamen **K** stamen **L** lodicules **M** ovary. Drawn from the holotype collection (*Peterson, Romaschenko & Valdés Reyna 24469*).

#### Distribution.

The new species is known from the Municipio de Bustamante in Tamaulipas, the Municipio Catorce in San Luis Potosí, the Municipio de Saltillo in Coahuila, and the Municipios Galeana and Santa Catarina in Nuevo León.

#### Conservation status.

The species is rare in México, but with more collecting it probably will be found in the adjacent state of Zacatecas.

#### Etymology.

The specific epithet honors Jesus Valdés Reyna (1948–), a renowned Mexican agrostologist, friend, and colleague who PMP, RJS, and KR have worked with for more than 35 years.

#### Ecology.

The new species has been found on calcareous (gypsum) rocky slopes and hillsides at 1700–2721 m associated with *Pinuscembroides* Zucc, *P.teocote* Schltdl. & Cham., *Juniperuscoahuilensis* (Martínez) Gaussen ex R.P. Adams, *Quercuspringlei* Seemen, *Cowaniamexicana* D. Don, *Rhusvirens* Lindh. ex A. Gray, *Partheniumincanum* Kunth. *Arctostaphylospungens* Kunth, *Arbutusxalapensis* Kunth, *Buddlejatomentella* Standl., *Bauhiniaramossisima* Benth. ex Hemsl., *Cercocarpusbetuloides* Nutt., *Yuccacarnerosana* (Trel.) McKelvey, *Agavelechuguilla* Torr., *A.gentryi* B. Ullrich, *Mimosabiuncifera* Benth., *Eriocomalobata*, *Muhlenbergia*, *dubia* E. Fourn., *M.pubescens* (Kunth) Hitchc., *Pseudoeriocomamultinodis* (Scribn. ex Beal) Romasch., *Erioneuronavenaceum* (Kunth) Tateoka, *Aristidacurtifolia* E. Fourn., *Boutelouauniflora* Vasey, *Berberis* sp., *Brickellia* sp., *Prunus* sp., *Salvia*, spp., *Stevia* sp., *Tagetes* sp., *Dasylirion* sp., *Ephedra* sp., *Brahea* sp., and *Heliotropium* sp.

#### Discussion.

Another species morphologically similar to *E.valdesii* in having sharp-pointed calluses, although not yet collected in México, is *E.scribneri* (Vasey) Romasch. found in the USA in western Texas, New México, Arizona, Colorado, Utah, and southeastern Wyoming (Barkworth 2007). However, *E.scribneri* differs in having shorter ligules ≤ 1.5 mm long with truncate apices and paleas 2.5–3.5 mm long (verses paleas 4–5 mm long in *E.valdesii*). *Eriocomaarida* (M.E. Jones) Romasch., also with sharp-pointed calluses, differs from our new species in having lemmatal awns 40–80 mm long that are obscurely 1-geniculate and scabrous throughout (Barkworth 2007). Reports of *E.arida* from Hidalgo and Nuevo León, México (Sánchez-Ken 2018) are perhaps in error since Dávila et al. (2018) did not record this species and Barkworth (2007) reported it as not found in México. *Eriocomaperplexa* (P.S. Hoge & Barkworth) Romasch. was reported in Dávila et al. (2018) and Sánchez-Ken (2018) as occurring in Coahuila but it was not included in Valdés Reyna's (2015), Gramíneas de Coahuila. It differs from *E.valdesii* in having shorter ligules 0.2–3.5 mm long [verses (2–) 4.5–8.5 mm long in *E.valdesii*] and a blunt callus only 0.4–0.6 mm long (verses 1–1.4 mm) [Barkworth 1993, 2007]. The new species can sometimes be confused with *Eriocomarobusta* (Vasey) Romasch., a much taller species up to 2.3 m tall with hairy collars, particularly on the flag leaves (glabrous or sparsely pubescent in *E.valdesii*), florets with a blunt callus, and lemmatal awns that are 20–32 mm long with the lower two segments scabrous (lemmatal awns 12–24 mm long and lower two segments short hairy in *E.valdesii*) [Barkworth 2007; Valdés Reyna 2015]. The new species is most similar to *Eriocomaalta* (Swallen) Romasch. since both species share long ligules, long leaf blades up to 60 cm long, and florets with sharp-pointed calluses (Swallen 1943). However, *E.alta* differs from the new species in having dark brown (verses straminious to light brownish in *E.valdesii*) florets 4–5.5 mm long (verses 6–7.5 mm long) with awns 8–12 mm long (versus 12–24 mm long), the awns scaberulous below (verses short hairy below), and short upper glumes 7–8 mm long (verses 8–12 mm long). *Peterson, Saarela & Romaschenko 23219* from Nuevo León differs from other collections of *E.valdesii* in having short, lacerate ligules about 2 mm long.

In our preliminary molecular DNA sequence analysis of most American species of *Eriocoma* there is a strongly-supported *E.lobata* I clade (including the type) found allied with *E.coronata* (Thurb.) Romasch., *E.parishii* (Vasey) Romasch., and *E.perplexa* (Valdés Reyna et al. 2013). In another portion of our tree a strongly-supported clade of five accessions of *E.valdesii* (including *Peterson, Romaschenko & Valdés Reyna 24469*, the type collection) forms a trichotomy with two other strongly-supported clades containing three accessions of *E.alta* (including the type) and seven accessions of *E.lobata* II (Valdés Reyna et al. 2013). All specimens examined below were sampled, except *Hoge, Barkworth & Valdés Reyna 295*, and formed a clade in our DNA analysis with the type.

#### Specimens examined.

México. **Coahuila**: Municipio de Saltillo, Highway 54 from Saltillo to Concepción del Oro, S to just past Estación Carneros, take road on right to tower, 2160 m, 18 Sep 1989, P.S. Hoge, M.E. Barkworth & J. Valdés Reyna 295 (ANSM, UTC); Sierra Madre Oriental, Estación Carneros, carretera 54, camino a la torre de microondas. 25°12'29"N, 101°24'01"W, 2258 m, 8 Sep 2008, Valdés Reyna & M.E. Barkworth 3085, 3087 (ANSM); 3 km above Estacion Carneros on road to radio tower, 25.12190N, 101.12006W, 2315–2400 m, 30 Oct 2019, *P.M. Peterson, K. Romaschenko & J. Valdés Reyna 26818* (CIIDIR, US). **Nuevo León**: Municipio Galeana, 2.3 mi N of Hwy 31 on dirt road, 24.70478N, 100.16019W, 2222 m, 9 Sep 2010, P.M. Peterson, J.M. Saarela & K. Romaschenko 23219 (US, CIIDIR); 27 km SW of Galeana on Hwy 58 towards San Roberto, 24.68214N, 100.11637W, 2 Nov 2019, *P.M. Peterson & K. Romaschenko 26884* (CIIDIR, US); Municipio Santa Catarina, edge of Parque Nacional Cumbres de Monterey, slopes above Puerto del Canejo, 25.49686N, 100.58644W, 2538–2721 m, 6 Sep 2010, P.M. Peterson, J.M. Saarela, K. Romaschenko & I. Cabral Cordero 23158 (US, CIIDIR). **San Luis Potosí**: Municipio Catorce, 3km E of San Jose de Coronados, 23.59105N, 100.89556W, 6 Nov 2019, *P.M. Peterson & K. Romaschenko 26941* (CIIDIR, US). **Tamaulipas**: Municipio de Bustamante, 16 km al SE de Bustamante hacia La Presita y Tula. 23°21'N, 99°40'W, 1700 m, 26 May 1982, J. Valdés Reyna & M.A. Carranza 1474 (ANSM, US-3103546).

### Key to the species of *Eriocoma* in northeastern México

**Table:** 

1	Callus sharp-pointed; ligules apices acute to acuminate, often lacerate, (2–) 4.5–8.5 mm long	**2**
–	Callus blunt and obtuse; ligules apices truncate, entire usually ≤ 2 mm long (rarely up to 3 mm)	**3**
2	Florets dark brown, 4–5.5 mm long; lemmatal awns 8–12 mm long, scaberulous below; upper glumes 7–8 mm long	***E.alta***
–	Florets straminious to light brown, 6–7.5 mm long; lemmatal awns 12–24 mm long, the awns short hairy below; upper glumes 8–12 mm long	***E.valdesii***
3	Flag leaves with a densely pubescent collar, the hairs 0.5–2 mm long; apex of lemma entire, not lobed; lemmatal awns 20–32 mm long	***E.robusta***
–	Flag leaves with a glabrous or sparsely pubescent collar; apex of lemma lobed, the lobes (0.3–) 0.5–1.2 mm long; lemmatal awns 8–18 (–22) mm long	***E.lobata* (I clade)**

## Supplementary Material

XML Treatment for Eriocoma valdesii
